# Les exostoses de Turrett’s: à propos de 35 cas

**DOI:** 10.11604/pamj.2018.29.229.14165

**Published:** 2018-04-26

**Authors:** Mounira Khezami, Achraf Abdennadher, Hiba Bellaaj, Talel Znagui, Mounir Hamdi, Lotfi Nouisri

**Affiliations:** 1 Service d’Orthopédie Traumatologie Hôpital Militaire Principal d’Instruction de Tunis, Tunisie

**Keywords:** Exostose, hallux, pied, chirurgie, Exostosis, hallux, foot, surgery

## Abstract

Nous rapportons à travers une étude rétrospective faite de 35 cas d’exostose sous unguéale de l’hallux dite exostose de Turrett's colligée entre 1995 et 2015 au service d’Orthopédie Traumatologie de l’Hôpital Militaire Principal d’Instruction de Tunis les résultats de la prise en charge de cette pathologie faite d’adultes jeunes d’âge moyen de 29 ans avec un sexe ratio de 1,7. Le délai moyen de consultation est de 18 mois. Ce retard de consultation est expliqué par une errance diagnostique en rapport avec une ressemblance du tableau clinique avec celui d’un ongle incarné. Le diagnostic est toujours confirmé par une radiographie de face et de profil de l’orteil intéressé. Le traitement a consisté en une exérèse radicale de l’exostose soit à travers une large fenêtre unguéale soit par un abord latéro-unguéal. L’examen anatomopathologique est systématique. Il a permis de confirmer la bénignité de l’affection dans tous les cas. La guérison a été de règle dans tous les cas avec une reprise de l’activité antérieure en 2 mois en moyenne. Aucune récidive n’a été notée.

## Abstract (en)

We conducted a retrospective study of 35 patients with subungual exostosis of the hallux, also known as Turrett’s exostosis, in the Department of Orthopedics and Traumatology at the Senior Military Hospital of Instruction of Tunis over the period between 1995 and 2015. We here summarize the outcomes of patients treated for this disease. The average age of patients was 29 years, with a sex ratio of 1.7. The median consultation time was six months. This delay in consultation was caused by a diagnostic error due to clinical picture resemblance with ingrown nail. Diagnosis was always confirmed by frontal and profile X-ray of the involved hallux. Treatment was based on total resection of the exostosis either through large ungual window or by latero-ungual approach. Anatomo-pathological examination was performed systematically. It allowed to confirm the benignity of the disease in all cases. All patients recovered and returned to their previous activity, on average, in 2 months. No patient had a recurrence.

## Introduction

L’exostose sous unguéale ou exostose de Turrett’s décrite pour la première fois par DUPUYTREN en 1847 est une tumeur ostéocartilagineuse bénigne relativement rare d’étiologie inconnue qui affecte la phalange distale des orteils ou des doigts [1]. Elle est caractérisée par son potentiel de récidive [2] . Le diagnostic est confirmé par la radiographie standard et le traitement est toujours chirurgical.

## Méthodes

Il s’agit d’une étude rétrospective descriptive faite au Service d’Orthopédie Traumatologie de l’Hôpital Militaire Principal d’Instruction de Tunis entre 1995 et 2015. Tous les patients ont bénéficié d’une radiographie de l’hallux de face et de profil. Les résultats ont été évalués cliniquement sur la douleur et l’état local ainsi que la reprise d’un chaussage normal, et radiologiquement sur l’exérèse totale ou non de l’exostose et sur la récidive.

## Résultats

**Figure 1:**
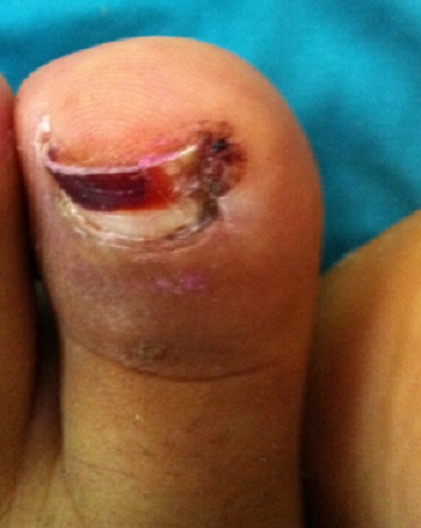
Aspect clinique de l’exostose de Turret's

**Figure 2:**
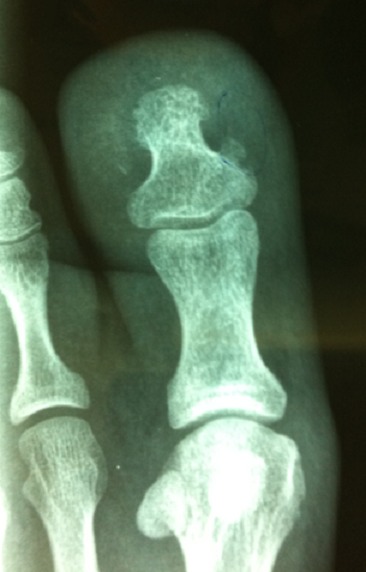
Radiographie de l’hallux de face montre une base d’implantation plus ou moins large de même tonalité osseuse que la phalangette

Notre série comporte 35 patients, il s’agit de 22 hommes et 13 femmes dont l’âge moyen est de 29 ans avec des extrêmes allant de 14 à 70 ans. Le tableau clinique est fait dans tous les cas de douleurs sous unguéales de l’hallux avec une dystrophie unguéale et gêne au chaussage simulant un pseudo tableau d’ongle incarné (Figure 1). Le délai moyen de consultation était de 14 mois et demi avec des extrêmes allant d’une semaine à 5 ans. Aucun facteur étiologique n’a pu être retenu en particulier la notion de microtraumatismes malgré que 19 patients de notre série fussent des militaires actifs portant des chaussures de combat. Le diagnostic a été presque toujours confirmé par une radiographie de l’orteil intéressé de face et de profil (Figure 2). Cependant la radiographie était normale chez 2 patients en rapport avec la non maturation de cette exostose.

**Figure 3:**
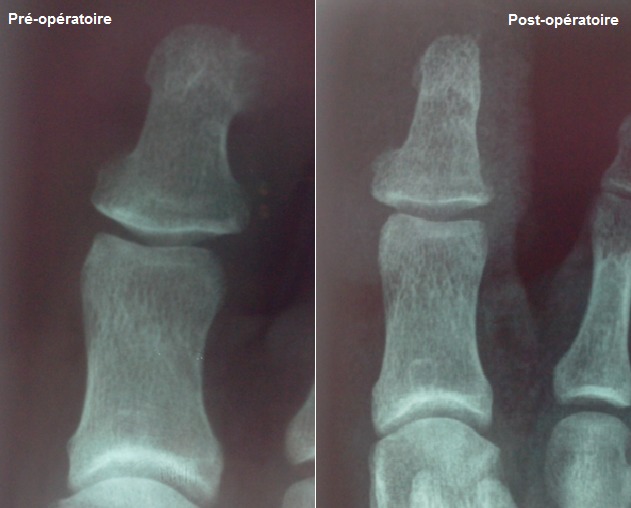
Résultat radiologique du traitement chirurgical

La Radiographie montre une base d’implantation plus ou moins large, de même tonalité osseuse que la phalangette, sans atteinte de cette dernière. Le traitement a été toujours chirurgical et a consisté en une exérèse radicale de l’exostose soit à travers une large fenêtre unguéale dans 19 cas ou par un abord latéral selon la localisation de l’exostose dans 16 cas (Figure 3). Le diagnostic d’exostose sous unguéale bénigne a été confirmé par l’examen anatomopathologique dans tous les cas. Au recul moyen de 6 mois et des extrêmes allant de 1 à 10 ans.

**Sur le plan clinique**: La cicatrisation cutanée a été obtenue en 3 à 4 semaines. Une infection post-opératoire a été décelée chez 2 patients qui ont bien évolué sous traitement antibiotique et soins locaux. La reprise de l’activité antérieure a été en moyenne de 2 mois.

**Sur le plan radiologique**: Aucune récidive locale et/ou une transformation maligne n’a été observée.

## Discussion

Les exostoses de Turrett’s sont relativement rares dans les populations orthopédiques générales et pédiatriques [3]. Actuellement, il y a peu de données probantes pour définir la démographie de ce groupe et orienter la gestion. C’est une tumeur ostéocartilagineuse dont l’étiopathogénie reste inconnue [4]. La plupart des hauteurs considéraient qu'il s'agissait d'une métaplasie réactive fibrocartilagineuse d’origine périostée résultant de microtraumatismes répétés [5]. L'infection, la tumeur, l'anomalie héréditaire ou l'activation d'un kyste cartilagineux ont été suggérés comme des étiologies possibles [6, 7]. Cette exostose sous unguéale prédominaient sur l’hallux comme le confirmaient plusieurs auteurs et posait un diagnostic différentiel avec un ongle incarné, un granulome pyogénique ou un mélanome sous unguéal [8], cependant le diagnostic devient aisé après une radiographie standard de l’hallux qui montrait une masse radio-opaque pédonculée sur la surface dorso-médiale de la phalange distale. Le traitement consistait en une exérèse chirurgicale marginale de l'exostose, qui atténuait généralement les symptômes et évitait la récidive [9]. Le rôle de la prise en charge non opératoire est limité car la maladie est progressive. D’après la littérature, la principale complication post-chirurgicale était la déformation de l'ongle qui est liée à la taille et à l'emplacement de la lésion lors de la présentation, une technique chirurgicale méticuleuse et une fermeture de la plaie pourraient minimiser ce risque [8].

## Conclusion

L’exostose sous unguéale reste une pathologie rare mais à connaître. Sur le plan diagnostique il faut faire la différence avec un ongle incarné et penser à faire une radiographie du gros orteil.

### Etat des connaissances actuelle sur le sujet

- L’exostose sous unguéale est une pathologie bénigne;
- Le tableau clinique simulait un pseudo tableau d’ongle incarné et le diagnostic est radiologique confirmé par l’examen anatomopathologique.

### Contribution de notre étude à la connaissance

- Aucun facteur étiologique n’est responsable de l’apparition de l’exostose de Turrett’s;
- Seul le traitement chirurgical par exérèse complète de l’exostose évitait la récidive de la maladie.

## Conflits d’intérêts

Les auteurs ne déclarent aucun conflit d'intérêts.
